# Targeting Latent Tuberculosis: Immunogenic Evaluation of a *Mycobacterium tuberculosis* Dormancy Antigen as a Vaccine Candidate

**DOI:** 10.3390/ijms27177827

**Published:** 2026-09-01

**Authors:** Rocío Zuazo, Ana Julia Bazán Bouyrie, Agustín Daniel Vitti, María Paula Morelli, Candela Martin, Javier Santos, Rosa Musella, Domingo Juan Palmero, Gabriela Calamante, María Paula Del Médico Zajac, Nicolás Oscar Amiano, Verónica Edith García

**Affiliations:** 1 Instituto de Química Biológica, Facultad de Ciencias Exactas y Naturales (IQUIBICEN), Universidad de Buenos Aires (UBA)-CONICET, Intendente Güiraldes 2160, Pabellón II, 4°piso, Ciudad Universitaria, Buenos Aires C1428EGA, Argentina; rociozuazo@qb.fcen.uba.ar (R.Z.); vittiagustin@gmail.com (A.D.V.); maria.paula.morelli@gmail.com (M.P.M.); candemartiin@gmail.com (C.M.); nicolasamiano@hotmail.com (N.O.A.); 2 Departamento de Química Biológica, Facultad de Ciencias Exactas y Naturales, Universidad de Buenos Aires (UBA), Intendente Güiraldes 2160, Pabellón II, 4°piso, Ciudad Universitaria, Buenos Aires C1428EGA, Argentina; ana.bazan19@gmail.com (A.J.B.B.); javiersantosw@gmail.com (J.S.); 3 Instituto de Biociencias, Biotecnología y Biología Traslacional (IB3), Facultad de Ciencias Exactas y Naturales, Universidad de Buenos Aires (UBA), Intendente Güiraldes 2160, Pabellón II, 2°piso, Ciudad Universitaria, Buenos Aires C1428EGA, Argentina; 4 División de Tisioneumonología, Hospital F.J. Muñiz, Uspallata 2272, Buenos Aires C1282AEN, Argentina; rmusella@intramed.net (R.M.); djpalmero@intramed.net (D.J.P.); 5 Instituto de Agrobiotecnología y Biología Molecular (IABIMO), Instituto Nacional de Tecnología Agropecuaria (INTA), Hurlingham, Buenos Aires C1033AAE, Argentina; calamante.gabriela@inta.gob.ar (G.C.); delmedicozajac.maria@inta.gob.ar (M.P.D.M.Z.); 6 Departamento de Química Orgánica, Facultad de Ciencias Exactas y Naturales, Universidad de Buenos Aires (UBA), Intendente Güiraldes 2160, Pabellón II, 3°piso, Ciudad Universitaria, Buenos Aires C1428EGA, Argentina

**Keywords:** tuberculosis, Rv2626c, immunogenicity, vaccines

## Abstract

An estimated 2.1 billion people are infected with *Mycobacterium tuberculosis* (*Mtb*) and at risk of developing active tuberculosis (TB). Because *Mtb* exists in replicating and dormant states, effective vaccines should target antigens from both stages. Here, we evaluated the immunogenicity and vaccine potential of *Mtb* dormancy antigen Rv2626c. Cellular and humoral immune responses were studied in individuals with latent TB infection (LTBI), active TB, and healthy donors (HD) using flow cytometry and ELISA. Overlapping peptides spanning Rv2626c sequence and structural mapping were employed to characterize Rv2626c immunogenic regions. Additionally, the protective efficacy of a Modified Vaccinia Ankara (MVA) vector expressing Rv2626c was evaluated in a murine challenge model. Rv2626c induced cellular immunity selectively in LTBI, characterized by increased frequencies of CD4^+^IFN-γ^+^, SLAMF1^+^, and poly-functional T lymphocytes. Immunodominant peptide regions were identified across the protein sequence. Moreover, mice immunized with MVA-Rv2626c showed a significant reduction in splenic bacterial burden following a challenge with *Mtb* H37Rv. Altogether, our findings indicate that the latency-associated antigen Rv2626c elicits immune responses in LTBI subjects and limits bacterial dissemination in our mice model of infection. Therefore, Rv2626c arises as a promising candidate to be combined with active phase antigens in multistage vaccines against *Mtb* infection.

## 1. Introduction

Tuberculosis (TB) caused by *Mycobacterium tuberculosis* (*Mtb*) remains a major global health problem, causing nearly 10 million new cases annually and representing the primary cause of mortality by a single pathogen worldwide. TB has always correlated with poverty, especially in low and middle-income countries. However, the incidence of TB has increased even in industrialized countries, mainly in vulnerable groups [1]. Furthermore, the strategies for TB control have been hampered by HIV co-infection and the emergence of drug-resistant *Mtb* strains [2]. TB infection encompasses a continuous spectrum of metabolic activity and immunological responses, with clinical outcomes that go from latent infection (defined by asymptomatic immune reactivity to the pathogen) to active disease (characterized by clinical manifestations). Additionally, the incipient and subclinical TB states (asymptomatic with very slight TB symptoms) were also added to the TB spectrum [3]. Host immune dynamics play a pivotal role in TB pathogenesis, influencing clinical progression. In fact, a primary challenge in controlling *Mtb* infection arrives from the ability of latent bacilli to persist in a viable state for decades before reactivating into active disease. The probability of transitioning from latency to active disease is higher during the initial five years following exposure, with pediatric and immunocompromised cohorts facing the maximum vulnerability. One-quarter of the world’s population is estimated to have been infected by *Mtb* [2]. These challenges show the need for improved preventive strategies, including vaccines capable of targeting both active and latent infection.

New TB vaccines that have entered clinical trials are based on *Mtb* antigens expressed during active bacterial replication. Thus, these preparations might prevent active *Mtb* infection but could not avoid reactivation of latent infection. Since the infecting bacteria consistently comprise dynamic subpopulations of replicating and dormant bacilli that can interconvert, ideal vaccines must protect against both replicating and dormant bacteria. Consequently, novel subunit vaccines should incorporate antigens representative of the two different phases of *Mtb* infection [4]. Actually, vaccines containing latency *Mtb* antigens might overcome this deficiency. Accordingly, Rv2626c (Hypoxic Response Protein 1; HRP1), a highly immunogenic DosR-regulated latency antigen, has emerged as a promising vaccine candidate [4]. Liu et al. used a construct integrated by antigens expressed by proliferating bacteria (ESAT6, peptide 190–198 of MPT64, and *Mtb*8.4) plus the dormancy antigen Rv2626c to make a multistage protein vaccine that generated antigen-specific immunity and induced high protective efficacy in a mouse model [4]. Moreover, a novel multiepitope vaccine containing epitopes from various *Mtb* latency antigens -including Rv2626c- was shown to induce robust immune responses in silico and in vitro and proposed as a vaccine candidate for the prevention of LTBI [5]. Interestingly, other prototypes of TB vaccines also containing Rv2626c have been suggested by using exosome-based delivery systems as a promising platform to develop efficacious TB vaccines to prevent latent *Mtb* infection [6].

Impaired immunity in *Mtb* infection is associated with diminished T cell activation and reduced production of IFN-γ [7,8]. Previously, we demonstrated that individuals latently infected with *Mtb* (LTBI) produce significantly higher levels of IFN-γ against Rv2626c as compared to TB patients and healthy donors [9]. Moreover, we further extended those results to analyze the cellular and humoral immune response to Rv2626c in a large population of individuals exposed for different periods of time to *Mtb*. Interestingly, our results showed that immune responses to the Rv2626c antigen allows to discriminate subjects with established latent *Mtb* infection from individuals recently exposed to the pathogen [10].

In the present work, we further investigated the immunogenicity of this dormancy antigen as a potential candidate for inclusion in multistage vaccines. To this end, we studied the polyfunctionality of the T-cell response against Rv2626c in individuals with LTBI, characterized by their effective immunity against *Mtb*. Moreover, we analyzed antigen-specific cellular and humoral immune responses at the single peptide level, identifying immunodominant regions and mapping them onto the three-dimensional structure of the protein. Finally, we evaluated the protective potential of Rv2626c delivered through a Modified Vaccinia Ankara (MVA)-based vaccine platform in a murine challenge model. By integrating peptide-level immune profiling, structural mapping, and in vivo vaccine evaluation, this study provides a comprehensive assessment of Rv2626c immunogenicity and its potential implications for the development of multistage tuberculosis vaccines.

## 2. Results

LTBI is associated with a dormancy state of the pathogen, during which several antigens are upregulated [11]. Previously, we studied the response of healthy subjects, LTBI individuals and TB patients to several DosR regulon-encoded antigens, including Rv2624c, Rv2626c and Rv2628 [9]. Interestingly, our previous results demonstrated that Rv2626c protein induced the secretion of significant amounts of IFN-γ from LTBI individuals, in sharp contrast to non-infected individuals and TB patients [9]. Therefore, we decided to analyze the immunogenicity of Rv2626c and investigate its potential as a vaccine antigen. For the human studies, three groups of individuals were recruited: LTBI subjects, TB patients and healthy donors (HD). Table 1 shows the demographic characteristics of the individuals included in this study.

### 2.1. Rv2626c Induces a Robust Antigen-Specific CD4^+^ T-Cell Response in LTBI Individuals

**Figure 1:**
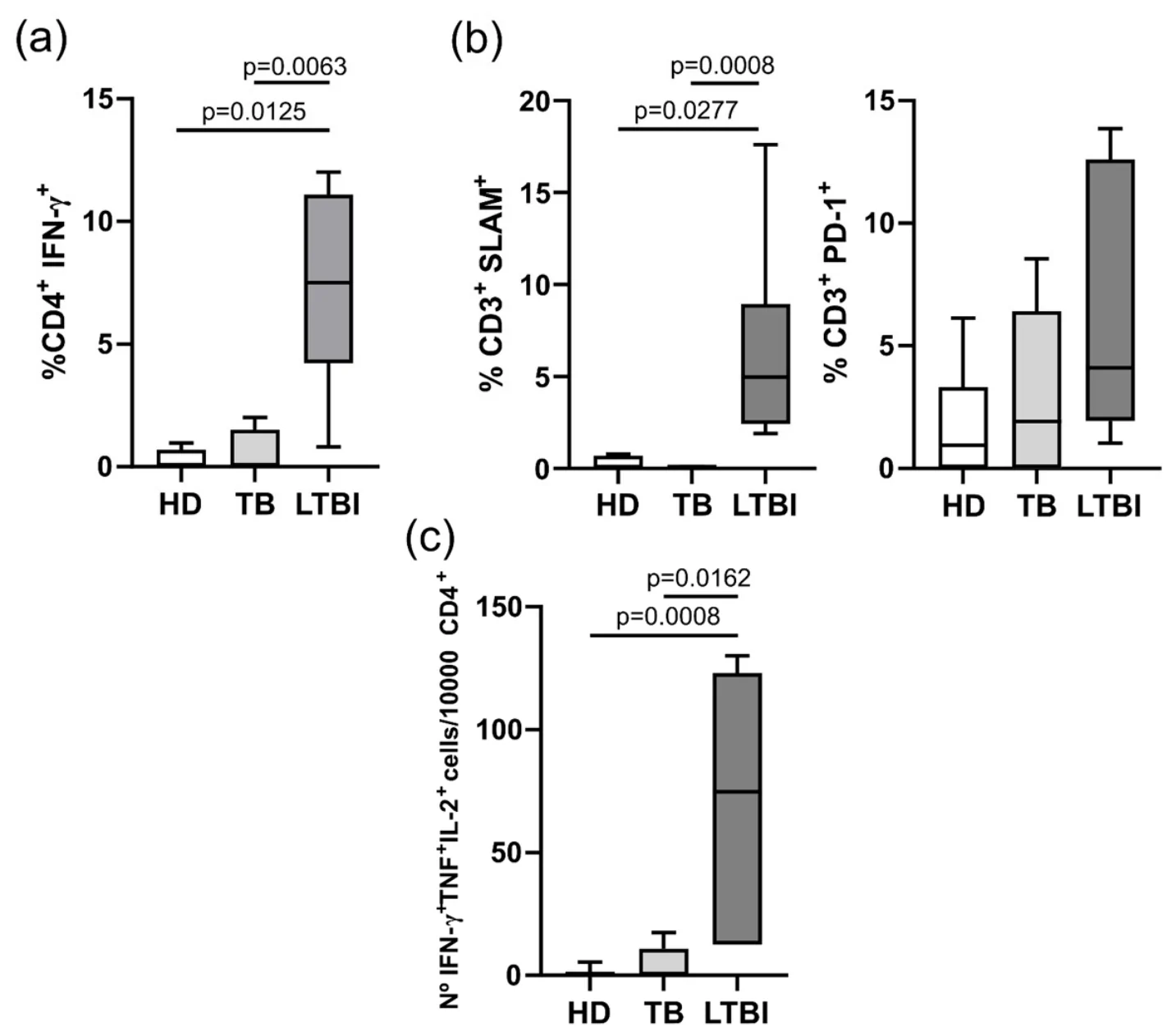
Characterization of the cellular immune response to Rv2626c by Flow cytometry. PBMCs were isolated from healthy donors (HD), individuals with latent tuberculosis infection (LTBI), and patients with active tuberculosis (TB), and cultured for 5 days in the presence or absence of recombinant Rv2626c protein (2.5 µg/mL). (**a**) Frequency of IFN-γ-producing CD4^+^ T cells in HD (*n* = 5), TB (*n* = 11), and LTBI (*n* = 7) individuals. Gating strategy is shown in Supplementary Figure S2. (**b**) Expression of the co-stimulatory molecule SLAMF1 (CD150) and the inhibitory receptor PD-1 (CD279) on lymphocytes from HD, TB, and LTBI individuals. Sample sizes were *n* = 6, 7, and 6 for SLAMF1 (CD150), and *n* = 11, 11, and 5 for PD-1 (CD279), respectively. Gating Strategy is shown in Supplementary Figure S3. (**c**) Frequency CD4^+^ T lymphocytes simultaneously producing IFN-γ, TNF-α, and IL-2 (per 10.000 CD4^+^ T cells) (*n* = 7/group). Gating strategy is shown in Supplementary Figure S2. Data are presented as box-and-whisker plots, with the box indicating the interquartile range (25th–75th percentiles), the center line indicating the median, and the whiskers indicating the minimum and maximum values. Statistical analysis was performed using the Kruskal–Wallis test followed by Dunn’s multiple-comparisons test with Bonferroni correction. A *p* value < 0.05 was considered statistically significant.

To further investigate the mechanisms underlying the Rv2626c-induced cellular immune response in LTBI subjects, we first evaluated antigen-specific T-cell activation. Effective immunity against *Mtb* infection is critically dependent on CD4^+^ Th1 lymphocytes, cells that play a critical role in granuloma formation and clearance of *Mtb* infection [12]. Given that our previous studies on IFN-γ production to *Mtb* latency antigens were performed by ELISA [9], in this work we investigated the cell mediated immunity (CMI) induced by Rv2626c by using flow cytometry. As shown in Figure 1a, LTBI individuals exhibited the highest frequencies of IFN-γ^+^ CD4^+^ lymphocytes following stimulation with Rv2626c, whereas TB patients and HD displayed markedly lower responses.

Furthermore, considering that co-signals are critical in T cell activation to mount effective antigen-specific immune responses, we next analyzed the expression of co-stimulatory and inhibitory molecules. Since we observed significant high numbers of IFN-γ^+^ lymphocytes against Rv2626c in LTBI subjects (Figure 1a), we studied whether SLAMF1 and PD-1 expression participated in T cell activation following Rv2626c stimulation. Remarkably, we observed significantly higher percentages of SLAMF1^+^ lymphocytes in LTBI compared to TB patients and healthy donors (Figure 1b, left). We also analyzed PD-1 expression in response to Rv2626c stimulation but no significant differences in the expression of this molecule was detected among the study groups (Figure 1b, right). Thus, the association between IFN-γ secretion and SLAMF1 expression suggests that T cells from LTBI subjects undergo substantial activation in response to Rv2626c antigen.

To further characterize the activation of T cells against Rv2626c, we analyzed the production of other critical cytokines that participate in CMI against *Mtb*. Then, we evaluated the simultaneous production of IFN-γ, TNF, and IL-2 in cells from LTBI individuals, TB patients, and healthy donors following Rv2626c stimulation. LTBI individuals exhibited markedly higher frequencies of polyfunctional IFN-γ^+^TNF^+^IL-2^+^ CD4^+^ T cells, while only minimal or undetectable responses were detected in TB patients and healthy donors (Figure 1c). Consequently, Figure 1 represents the results obtained with Rv2626c that displayed noticeable differences among individuals and highlights the ability of T cell activation in LTBI subjects by this dormancy antigen.

### 2.2. Identification of Immunodominant Rv2626c Regions Associated with Cellular and Humoral Immunity

**Figure 2:**
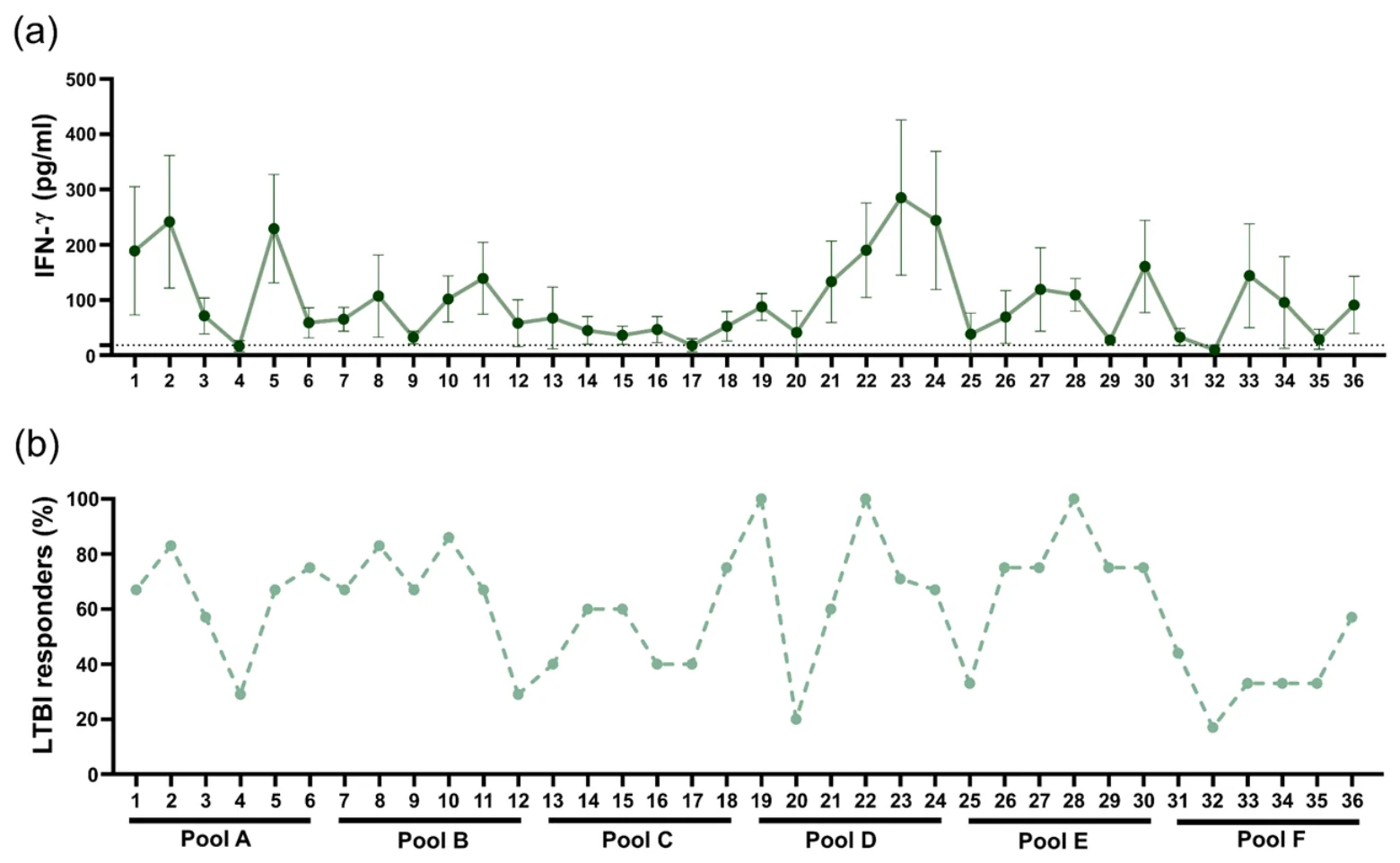
Peptide-specific IFN-γ responses to Rv2626c-derived peptides. (**a**) IFN-γ production against individual Rv2626c-derived peptides by PBMCs from LTBI subjects. Cells were stimulated with each individual peptide (5 μg/mL each), and IFN-γ levels in culture supernatants were quantified by ELISA after 5 days of incubation. Mean IFN-γ concentrations (pg/mL) ± SEM obtained from LTBI subjects are shown for the 36 overlapping peptides spanning the entire amino acid sequence of Rv2626c. The number of individuals analyzed varied among peptides (*n* = 4–9). The dotted line represents the operational threshold (18 pg/mL) and was used to define peptide-specific responder individuals. (**b**) Percentage of LTBI responders for each peptide, defined as the proportion of LTBI subjects displaying peptide-specific IFN-γ responses above the established threshold.

Previously, by using pools of overlapping peptides of Rv2626c, we identified the most immunogenic ones that allow to specifically differentiate LTBI subjects from TB patients and HD [9]. Here we analyzed the immunogenicity of individual peptides from the protein. To do this, PBMCs from LTBI subjects were stimulated with 36 overlapping synthetic peptides spanning the entire Rv2626c sequence (Supplementary Figure S1 and Supplementary Table S1) and IFN-γ production was determined. The results showed that 33 out of 36 peptides elicited mean IFN-γ levels above the operational threshold (18 pg/mL). The only exceptions were peptides 4, 17 and 32 (Figure 2a). The 18 pg/mL threshold used in the present study corresponds to 0.45 IU/mL of IFN-γ, which was previously established as the cutoff for an Rv2626c-based IGRA [10]. In our previous study, the 0.45 IU/mL cutoff was determined by ROC analysis and showed 78.95% sensitivity and 83.02% specificity for differentiating individuals with LTBI from other *Mtb*-exposed individuals and patients with active TB. It is important to mention that the previous assay used the recombinant Rv2626c protein as the stimulating antigen, whereas the present study used individual Rv2626c-derived peptides. Therefore, in this work, the 18 pg/mL (0.45 IU/mL) value was used as an operational threshold for exploratory peptide screening rather than as a peptide-specific diagnostic cutoff.

Importantly, the identification of immunodominant regions must consider not only the magnitude of the IFN-γ response elicited by each peptide, but also the proportion of responder subjects from the LTBI population. Thus, in addition to IFN-γ levels, we also analyzed the percentage of LTBI subjects who responded to each peptide by using the aforementioned threshold. As shown in Figure 2b, the percentage of LTBI responders for each peptide showed important differences. In fact, whereas several peptides elicited high levels of IFN-γ, only a small number of LTBI individuals recognized them as immunogenic peptides. Nevertheless, peptides 2, 5, 10, 11, 28, 30 and the region 21–24 not only elicited strong IFN-γ responses but were also recognized by the highest proportion of LTBI subjects (Figure 2b). Therefore, we were able to identify six immunodominant peptides, plus an immunodominant region of the Rv2626c protein, that generate a strong CMI in LTBI individuals.

To assess the potential influence of HLA class II restriction on the immunogenicity of the Rv2626c regions identified in this study, we considered the results of a previous in silico analysis performed in our laboratory using the same Rv2626c-derived peptides [13]. In that study, peptides 7–12, 19–24, and 31–36 were evaluated for their predicted binding to representative HLA class II molecules using NetMHCII. The resulting binding data were subsequently used to estimate population coverage with the IEDB Population Coverage tool. The analysis included 161 populations grouped according to geographic region and showed broad predicted population coverage across all regions analyzed. Importantly, six of the ten peptides identified as IFN-γ-reactive in the present study (peptides 10, 11, 21, 22, 23, and 24) were included in our previous analysis. These findings provide supportive evidence that a substantial proportion of the cellular immunogenic regions identified in the present study may have broad potential coverage across genetically diverse human populations.

**Figure 3:**
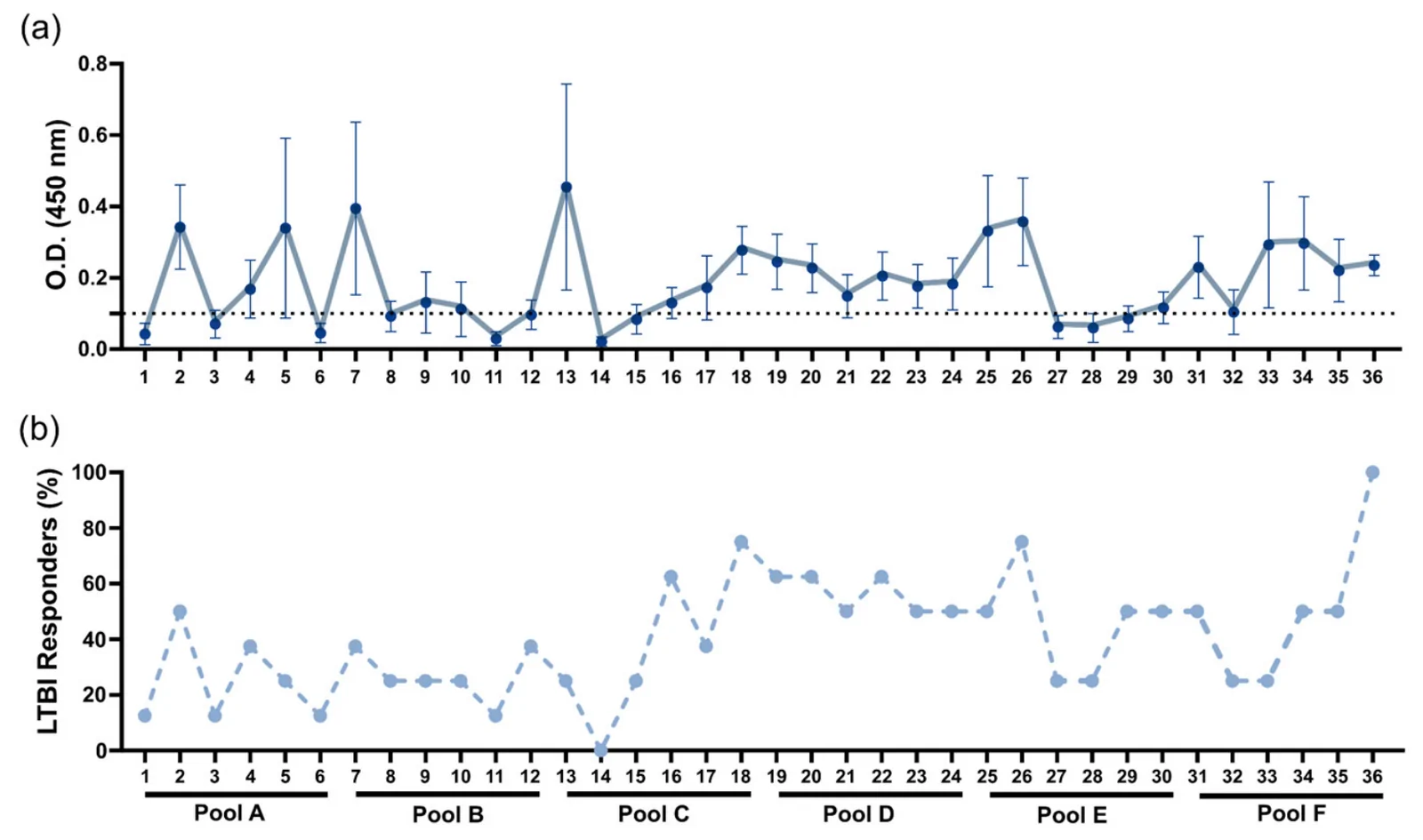
Peptide-specific IgG antibody responses to Rv2626c-derived peptides. (**a**) Levels of IgG antibodies against each Rv2626c-derived peptide in LTBI individuals. Antibody levels were determined by ELISA using each individual peptide to coat high-binding plates (2.5 μg/mL), leaving uncoated wells as control for unspecific binding. Mean optical density (O.D.) values at 450 nm ±-SEM obtained from plasma samples of LTBI individuals (*n* = 8) are shown for the 36 overlapping peptides covering the entire amino acid sequence of Rv2626c. The operational threshold (0.1 O.D.) was defined as the mean O.D. plus three standard deviations (mean + 3 SD) of Rv2626c peptide-specific IgG levels measured in 20 healthy donors (HD) (represented by a dotted line). (**b**) Percentage of responder individuals for each peptide, defined as the proportion of LTBI individuals with peptide-specific antibody levels above the established threshold.

In addition, we have reported increased plasma levels of Rv2626c-specific IgG antibodies in LTBI individuals [10]. Based on this, we determined the levels of specific circulating antibodies against each of the 36 overlapping peptides mentioned before, ELISA plates were coated with the individual peptides, and IgG levels in plasma of LTBI individuals were subsequently analyzed. Although several peptides elicited detectable IgG responses, peptides 16, 18, 19, 20, 22, 26, and 36 combined high IgG reactivity with the highest responder frequencies among LTBI individuals (Figure 3), identifying them as the major immunodominant regions associated with the humoral immune response to Rv2626c.

### 2.3. Structural Mapping of Identified Immunogenic Regions of Rv2626c

**Figure 4:**
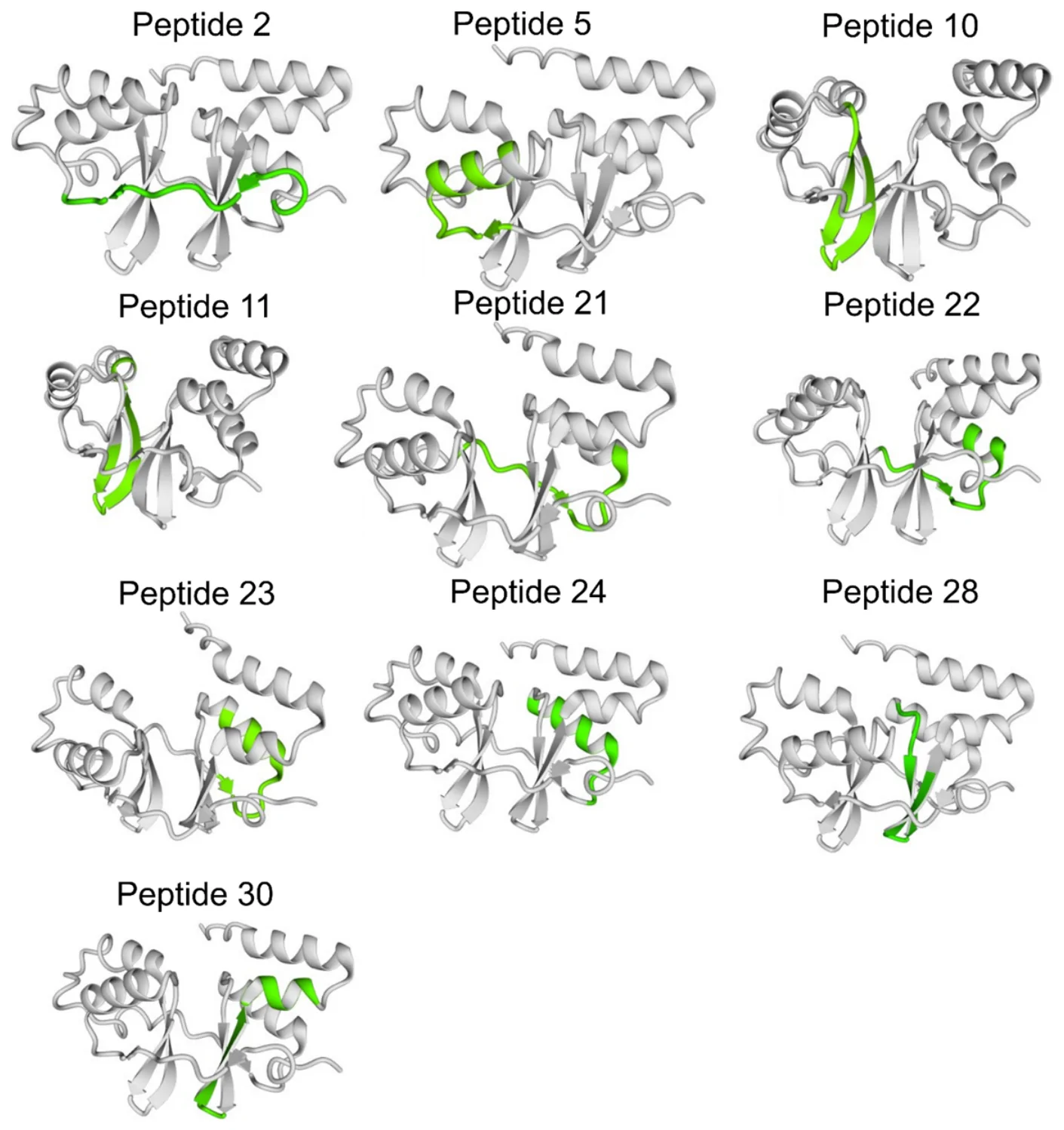
Three-dimensional representation of Rv2626c showing the immunogenic regions associated with specific IFN-γ responses, highlighted in light green. The structure was visualized using YASARA [14].

**Figure 5:**
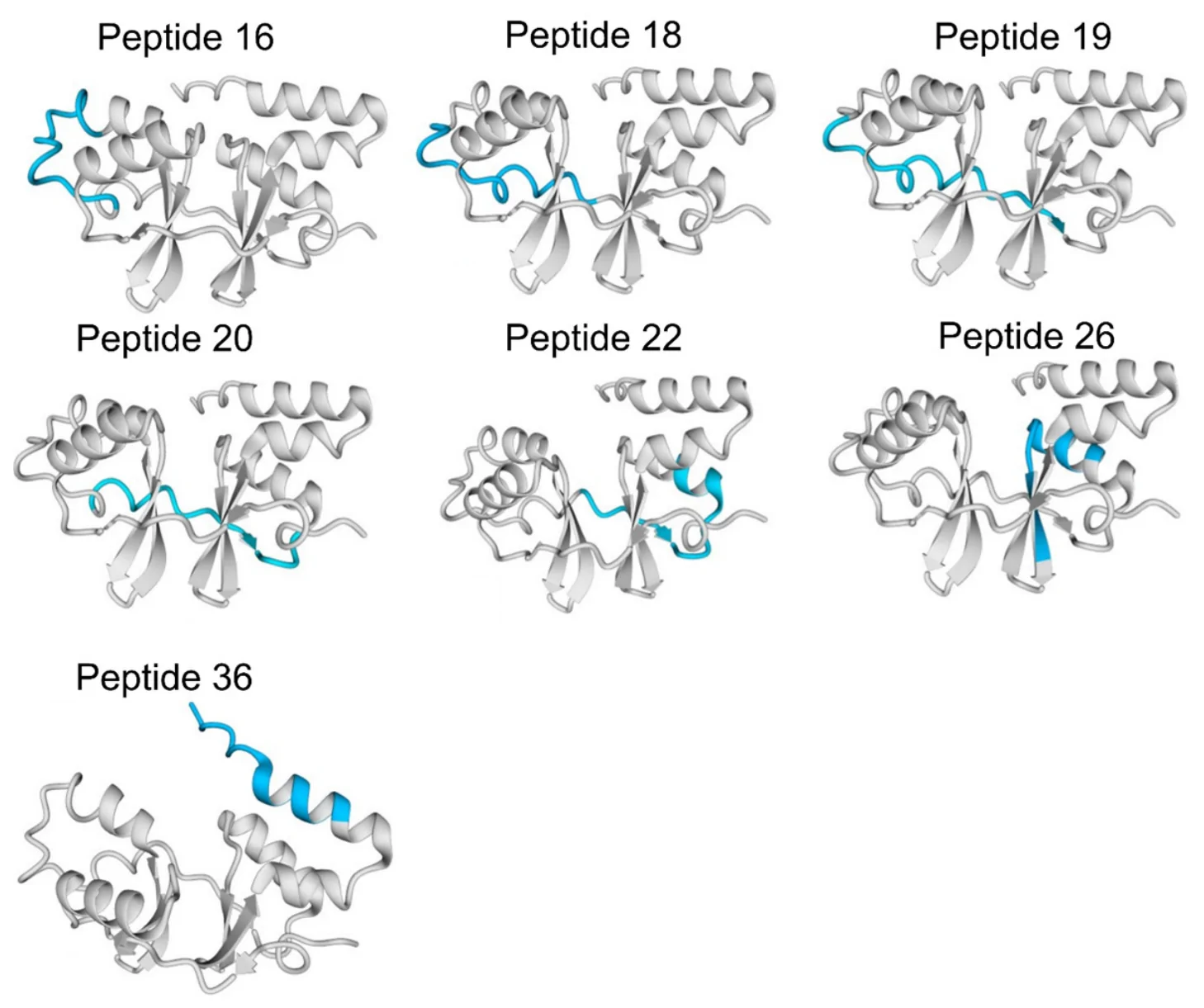
Three-dimensional representation of Rv2626c showing the immunogenic regions associated with specific antibody responses, highlighted in cyan. The protein structure was visualized using YASARA [14].

To further characterize the cellular and humoral immune response patterns associated with the distinct immunogenic regions identified, the location of these regions within the three-dimensional structure of Rv2626c was analyzed. The structural mapping was performed using immunogenic peptides from the IFN-γ and IgG analyses and it was used to visualize its distribution within the protein structure. The regions identified through the peptide-specific IFN-γ analysis are shown in light green (Figure 4), whereas those identified through the peptide-specific antibody analysis are shown in cyan (Figure 5).

As we mentioned before, the IFN-γ analysis identified peptides 2, 5, 10, 11, 21, 22, 23, 24, 28, and 30 as immunogenic regions of Rv2626c (Figure 4). These sections were distributed across different structural elements of the protein, including central β-strands and non-regular secondary-structure zones. In particular, the central β-strands forming part of the protein core were included among the regions associated with the IFN-γ response. The non-regular secondary-structure stretches located at residues 1–20 and 60–86 were also included among the experimentally identified IFN-γ-reactive regions. In contrast, the C-terminal α-helix was not included among the regions identified through the IFN-γ analysis.

Similarly, the peptide-specific IgG analysis experimentally identified seven immunogenic regions corresponding to peptides 16, 18, 19, 20, 22, 26, and 36 (Figure 5). Mapping these identified antibody-reactive peptides onto the protein structure showed that they were distributed across several structural elements. In particular, antibody-reactive sections encompassed α-helix 3 and the C-terminal α-helix (α-helix 5), as well as an exposed non-regular secondary-structure region corresponding to residues 60–86. On the other hand, only one of the four central β-strands was included among the regions identified by the antibody analysis, which may reflect the lower accessibility of these structural elements.

**Figure 6:**
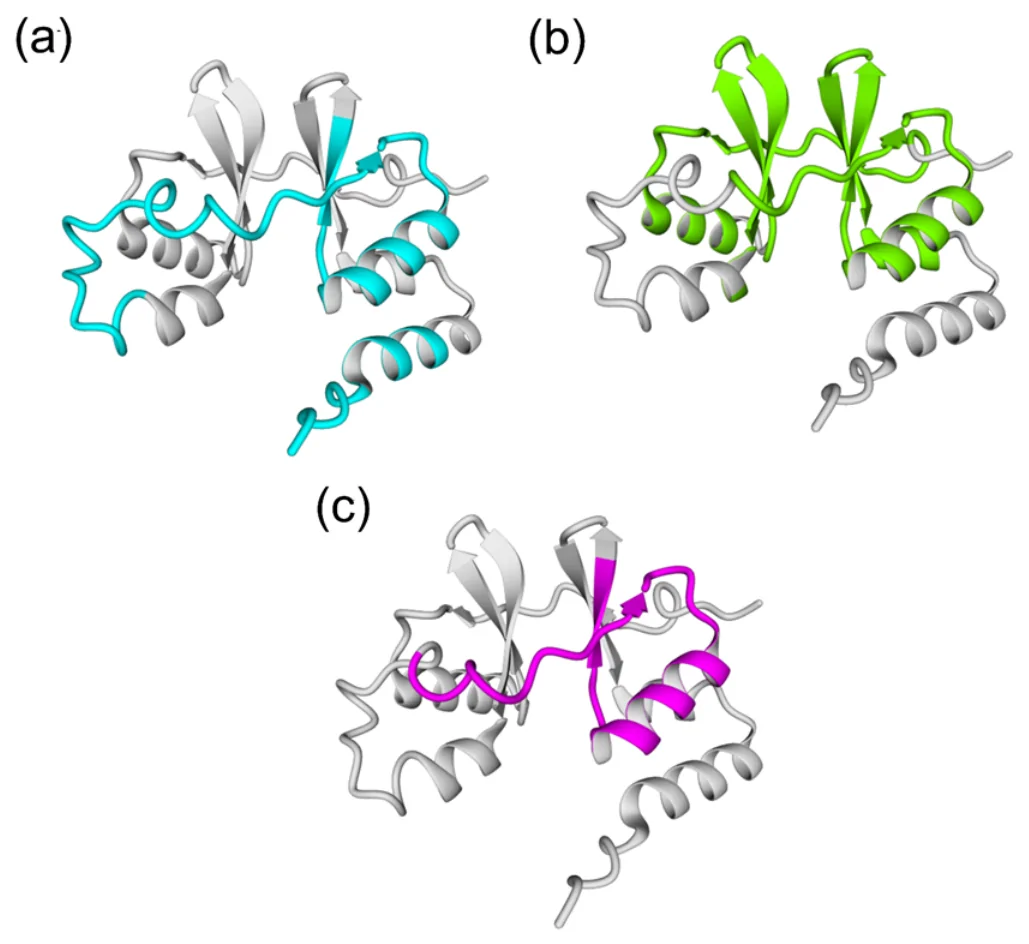
Summary of the immunogenic responses mapped onto the three-dimensional structure of Rv2626c. (**a**) Regions associated with antibody responses, highlighted in cyan. (**b**) Regions associated with IFN-γ responses, highlighted in light green. (**c**) Region identified by both antibody and IFN-γ analyses, highlighted in purple. The protein structure was visualized using YASARA [14].

Importantly, the structural mapping revealed an overlap between the regions identified through the cellular and humoral immune responses (Figure 6). Of the 143 residues comprising Rv2626c, 89 residues (62%) were included within the regions identified by the IFN-γ analysis, whereas 52 residues (36%) were included within the regions identified by the IgG analysis. Thirty-three residues (23%) were shared between the regions identified by both approaches. Remarkably, only 15% of residues were not involved in these responses. Among the peptide regions analyzed, peptide 22 (SIYYVDANASIQEML) was identified in both the IFN-γ and IgG analyses, further highlighting this region as a site with overlapping cellular and humoral immune recognition.

Overall, this structural representation contextualizes the experimentally identified immunogenic regions within the three-dimensional structure of Rv2626c and provides insights into their potential exposure to immune recognition, although experimental validation remains necessary to determine their actual accessibility and functional relevance.

### 2.4. MVA-Rv2626c Vaccination Reduces Bacterial Dissemination Following Mtb Challenge

**Figure 7:**
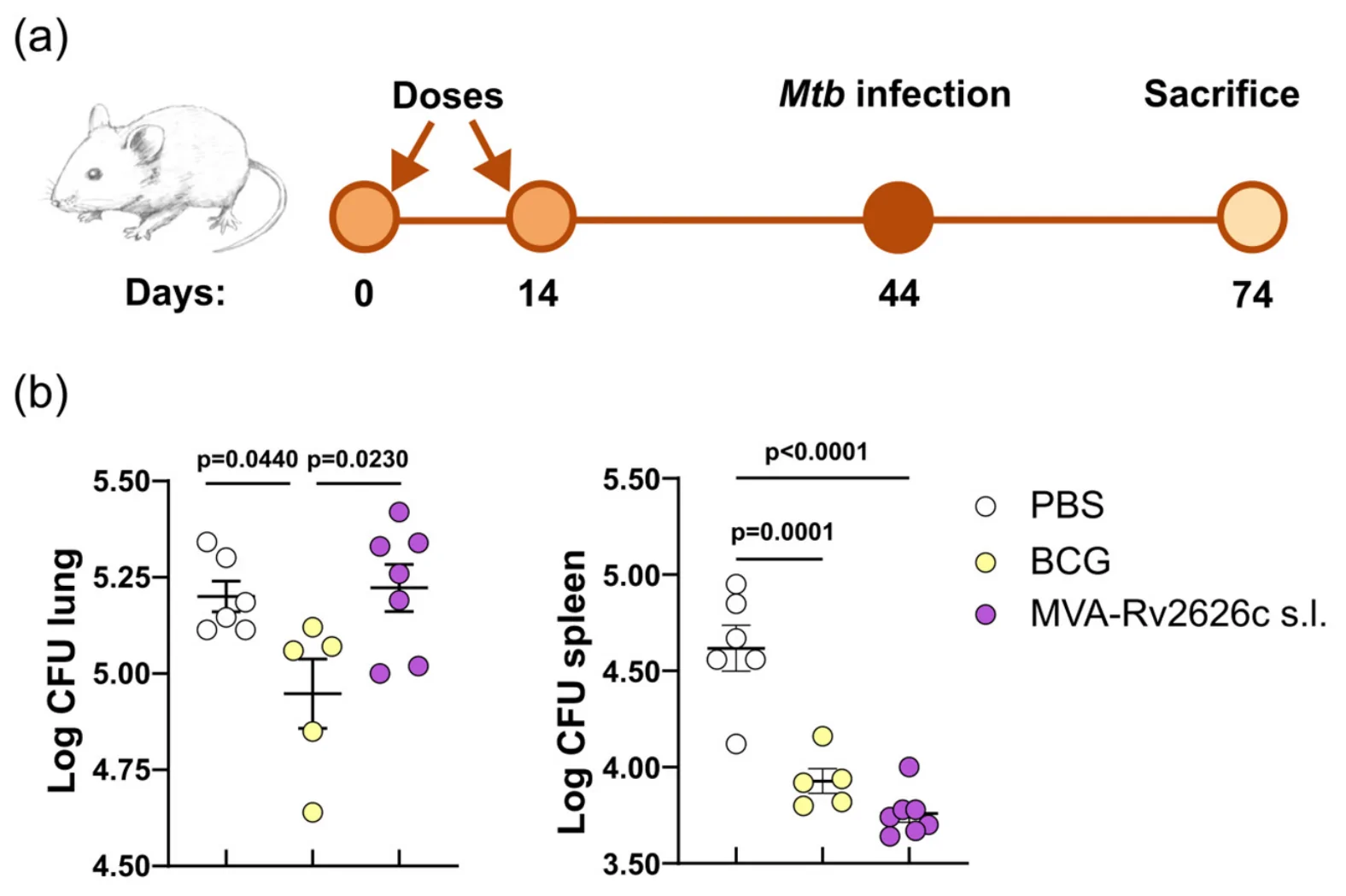
(**a**) Experimental schedule. Female BALB/c mice were immunized with two doses of MVA-Rv2626c at day 0 and 14. Four weeks after the last immunization, animals were challenged intratracheally with *Mtb* H37Rv. Animals were euthanized four weeks after the challenge. PBS (*n* = 6), BCG (*n* = 5), and MVA-Rv2626c (*n* = 7) groups were included. (**b**) Bacterial burden in the lungs and spleens was determined by culturing organ homogenates and enumerating colony-forming units per milliliter (CFU). Each dot represents the log CFU per organ from triplicate plate counts for an individual mouse. Log transformation was applied prior to analysis to satisfy the normality assumption. Comparisons among groups were performed using one-way ANOVA followed by Tukey multiple comparisons test. A *p* value < 0.05 was considered statistically significant.

The results obtained in LTBI individuals demonstrated that Rv2626c elicits antigen-specific cellular and humoral immune responses. Therefore, we next investigated the potentially protective response against this antigen in vivo using a murine model of tuberculosis previously established in our laboratory [15]. To do this, mice were immunized sublingually (s.l.) with a MVA vector expressing Rv2626c (MVA-Rv2626c) or with *Mycobacterium bovis* BCG Danish, (5 × 10^4^ CFU), following the experimental schedule shown in Figure 7a. Thirty days after the final immunization, mice were challenged with the virulent *Mtb* H37Rv strain, and bacterial burden was determined 30 days later by quantifying colony-forming units per milliliter (CFU) in the lungs and spleen. Animals immunized with PBS or BCG were used as negative and positive controls, respectively. As shown in Figure 7b (left), no significant differences in pulmonary bacterial burden were observed among PBS, and MVA-Rv2626c-immunized mice. In contrast, animals vaccinated with MVA-Rv2626c displayed a significant reduction in splenic bacterial burden compared with PBS-immunized controls (*p* < 0.01), indicating protection against systemic bacterial dissemination following *Mtb* challenge (Figure 7b, right).

We also studied the immune response induced by vaccination, evaluating the Rv2626c-specific cellular response. As shown in Supplementary Table S2, a significant increase in both the frequency of specific IFN-γ-producing CD4^+^ T cells and IFN-γ production was detected compared with the PBS group (*p* < 0.0001). Although no reduction in pulmonary bacterial burden was observed, the marked decrease in splenic bacterial burden induced by MVA-Rv2626c vaccination suggests a potential protective effect against systemic bacterial dissemination (Supplementary Table S3).

Overall, our findings show that MVA-Rv2626c immunization induces Rv2626c-specific cellular immune responses and is associated with a significant reduction in splenic bacterial burden following *Mtb* challenge. These findings support further investigation of Rv2626c as a potential latency-associated antigen for inclusion in a multistage vaccine against TB.

## 3. Discussion

Protection against *Mtb* requires T cell activation and IFN-γ production [16]. In fact, reduced IFN-γ-production by PBMCs is a marker of-severe disease [17], confirming the role of cell mediated immunity (CMI) in protection against tuberculosis (TB) [7]. Accordingly, we have previously demonstrated that High Responder TB patients displayed strong immune responses to *Mtb*, established by T cell activation and IFN-γ secretion against the pathogen. In contrast, Low Responder TB patients displayed weak or no T cell responses to *Mtb*, leading to advanced clinical severity. On the opposite side, most *Mtb*-infected individuals remain clinically asymptomatic and they do not develop active TB (LTBI), highlighting that CMI controls the infection in most of them [16,17]. However, the specific immune components responsible for preventing progression to active disease or reactivation remain incompletely understood. Therefore, characterization of immune responses associated with LTBI may provide useful information for the rational selection of antigens for multistage TB vaccine development.

Previously, we demonstrated that Rv2626c, a dormancy-associated protein encoded by the DosR regulon and highly expressed during latency [7], induces strong IFN-γ responses in whole blood and peripheral blood mononuclear cells from individuals with LTBI [9,10]. In this study, we deeply investigated the immunogenicity of this conserved protein by analyzing its ability to induce specific T cell activation, including the expression of costimulatory molecules, expansion of IFN-γ lymphocytes and polyfunctional T cells. These complementary approaches allowed us to investigate not only the magnitude but also the quality of the immune response associated with this dormancy antigen. Following Rv2626c stimulation, lymphocytes from LTBI subjects displayed higher SLAMF1 expression than those from TB patients and healthy donors (Figure 1b). In line with these results, we previously demonstrated that SLAMF1 expression contributes to Th1 cytokine responses in active TB [7]. Interestingly, it has been recently demonstrated that direct contact between macrophages and CD4^+^ T cells mediates protection to *Mtb* through the homotypic molecule SLAMF1 [18]. Our current data suggest an interesting association between SLAMF1 expression and an increased immune response in LTBI subjects. However, since no functional analyses were performed, the differential levels of SLAMF1 expressed in LTBI in response to Rv2626c stimulation do not establish a specific protective mechanism. Thus, additional investigations are warranted to uncover the underlying mechanisms driving this association.

Several studies in vaccine immunology and chronic viral infections have suggested that a polyfunctional cellular response -that is, cells that produce more than one cytokine simultaneously- would be more efficient in providing protection than responses primarily composed of cells that produce only one cytokine [19]. Based on the above, it was suggested that the degree of cellular polyfunctionality could also be used as a marker for monitoring treatment in LTBI individuals [20]. Therefore, we evaluated the ability of cells from LTBI individuals, HD, and TB patients to simultaneously secrete IFN-γ, TNF-α, and IL-2 in response to Rv2626c. As shown in Figure 1c, significantly higher numbers of IFN-γ^+^TNF^+^IL-2^+^ cells were detected among LTBI’s CD4 lymphocytes as compared to TB patients and healthy donors, suggesting that Rv2626c antigen induces the strongest T cell activation in LTBI subjects.

Previously we searched for the immunodominant regions of Rv2626c to which only LTBI responded. For this, we employed overlapping peptides arranged in pools composed of six peptides each, covering the entire Rv2626c protein and we were able to identify the most specific and immunogenic ones which could strongly differentiate among LTBI, TB patients and HD [9]. Therefore, we sought to investigate the individual peptides from Rv2626c that induced the highest IFN-γ levels, identifying at the same time, the percentages of LTBI subjects capable of responding to each peptide from this antigen (Figure 2). Interestingly, we were able to identify the peptide region 21 to 24 as the most immunogenic sequence of Rv2626c protein (Figure 2). In line with our findings, Naz et al. recently reported, using a semiempirical in silico approach, that Rv2626c is a highly antigenic target capable of eliciting Th1-type immune responses [6].

The magnitude of peptide-specific IFN-γ responses and the frequency of responders represent two related but distinct characteristics of peptide immunogenicity. Some peptides may induce strong IFN-γ responses in a subset of LTBI individuals while being poorly or not recognized by other individuals. Thus, a high magnitude of the response does not necessarily imply recognition by a large proportion of the LTBI population. This inter-individual variability could be influenced by HLA polymorphism, which can affect peptide processing and presentation and consequently the recognition of specific T-cell epitopes. In addition, differences in the individual T-cell repertoire and immune status may contribute to variability in the magnitude of responses among individuals capable of recognizing the same peptide. Therefore, both response magnitude and responder frequency should be considered complementary parameters when identifying candidate immunodominant regions of Rv2626c. A limitation of the present study is the relatively small size of the human cohorts. Therefore, the frequencies of responders observed in the LTBI cohort should not be considered representative of the broader LTBI population. Rather, the immunogenic regions identified here should be regarded as candidate regions requiring validation in larger, independent cohorts. Future studies, ideally including geographically and genetically diverse populations together with HLA characterization, will be important to assess the reproducibility and population-level relevance of the Rv2626c-specific immune responses identified in the present study. However, it is important to mention that although HLA typing was not performed in the individuals included in the present study, a previous in silico analysis performed in our laboratory using the same peptide sequences demonstrated broad predicted HLA class II population coverage, including six of the ten IFN-γ-reactive peptides identified here, supporting the potential recognition of these regions across genetically diverse populations [13]. Nevertheless, individual HLA restriction could not be directly assessed in the present cohort and should be addressed in future studies.

The protective role of antibodies after BCG vaccination has remained uncertain. Nevertheless, recent studies have contributed with evidence for their role in protection against *Mtb* infection, as biomarkers for the infection stage, and as predictors of outcomes [21]. Accordingly, we previously reported that anti-Rv2626c IgG levels are significantly higher in LTBI individuals than in healthy donors and TB patients [10]. Here we further investigated the levels of specific circulating antibodies in response to each of the 36 overlapping peptides spanning the entire Rv2626c sequence as well as the percentage of LTBI subjects recognizing each of them. In this way, we found that peptides 16, 18, 19, 20, 22, 26, and 36 were recognized by specific IgG antibodies in approximately 60% and 100% of the LTBI individuals tested against each respective peptide (Figure 3).

Although antibody responses are often associated with pathogens that persist extracellularly in the host, *Mtb* primarily resides within macrophages, which are key cellular components of pulmonary granulomas. From there, the infection can spread to other parts of the host, like spleen, kidneys, and brain [22]. Antibody signatures have been successfully used as biomarkers of disease, for example, to differentiate between latent and active TB [10] providing additional evidence for the model of humoral fluidity [21]. Advancements in understanding the role of antibodies to prevent *Mtb* infection are critical for the development of novel strategies to prevent reactivation and progression of TB disease. In fact, protective antibodies against *Mtb* infection could be used as potential standards for vaccine development, whereas humoral immunity induced by *Mtb* infection could be exploited for post-infection treatment [21].

To further characterize the immunogenic regions identified in our peptide-screening experiments, we mapped the IFN-γ- and antibody-associated peptides onto the three-dimensional structure of Rv2626c. The IFN-γ-associated regions included peptides 2, 5, 10, 11, 21, 22, 23, 24, 28, and 30, whereas antibody-associated regions included 16, 18, 19, 20, 22, 26, and 36. Interestingly, peptide 22 (SIYYVDANASIQEML) was identified in both analyses, suggesting that this region may contribute to both cellular and humoral recognition of Rv2626c. The remaining regions showed distinct patterns of recognition, indicating that cellular and humoral immune responses may target different structural regions of the protein. Mapping these regions onto the Rv2626c structure provides a framework for evaluating their structural context and potential accessibility to immune recognition. However, further experimental studies will be required to determine the precise contribution of each region to antigen processing, T-cell recognition, and antibody binding, while establishing their functional relevance in the immune response to Rv2626c.

Some of the immunogenic regions identified in the present study overlap with previously described Rv2626c T-cell epitopes in mice. Roupie et al. [23] performed an epitope-mapping analysis of Rv2626c using overlapping 20-mer peptides in DNA-vaccinated BALB/c and C57BL/6 mice and identified several immunodominant regions. Among these, the Rv2626c region spanning residues 71–90, was recognized in both mouse strains. Within this region, the sequence IYYVDANASI (residues 78–87) was further identified as an H-2K^d^-restricted CD8^+^ T-cell epitope capable of inducing IFN-γ production and cytotoxic activity. Interestingly, this region substantially overlaps with peptides 21, 22, and 23 in our present study, which were identified as immunogenic based on IFN-γ responses in LTBI individuals. In particular, peptide 22 encompasses the previously described Rv2626c 78–87 epitope and was identified in the present study as a target of both cellular and humoral immune responses, showing IFN-γ reactivity as well as IgG recognition in LTBI individuals. This finding highlights a region of Rv2626c that appears to be immunologically relevant in both human and murine systems. However, the correspondence is not complete, as other immunodominant regions identified by Roupie et al., located approximately within residues 121–140 [23], were not among the major IFN-γ-associated regions identified in our LTBI cohort. These findings highlight both the conservation and the potential differences in Rv2626c immune recognition between experimental animal models and humans.

During nearly 100 years, the attenuated *Mycobacterium bovis* Bacillus Calmette-Guérin (BCG) has been the only vaccine available to be administered in humans. Although BCG is more effective in protecting children against tuberculous meningitis and disseminated forms of TB [24,25], its protection against the pulmonary form of TB in adults remains highly controversial [26], particularly against reactivation of latent infection. Given that one quarter of the world population is latently infected with *Mtb* [2] there is an urgent need for the development of new vaccine strategies.

The Modified Vaccinia Ankara (MVA) vaccine vector expressing the mycobacterial antigen 85A (MVA85A) was demonstrated to be safe, although it did not improve BCG efficacy. Therefore, several strategies including heterologous prime/boost protocols, the use of co-stimulatory molecules, and the combined use of adjuvants [27] have been explored to augment MVA immunogenicity. In line with these strategies, Zhu et al. demonstrated that a vector hyperexpressing three costimulatory molecules (B7-1, ICAM-1, and LFA-3) with ligands on T cells can be used to efficiently infect human DCs, leading to enhanced peptide-specific T-cell activation [28]. Interestingly, in the present work, we demonstrated that the self-ligand SLAMF1, previously shown to prime a unique signaling pathway in T cells [29,30] was up-regulated in response to Rv2626c stimulation in LTBI subjects (Figure 1), denoting the potency of this antigen itself to induce activation of T lymphocytes. Interestingly, Prasad et al. demonstrated that SLAMF1 expression depends on CMI and autophagy as well [18]. Accordingly, we have reported that activation of SLAMF1 increases neutrophil autophagy induced by *Mtb* [31]. Given that this costimulatory molecule is increased after Rv2626c stimulation in LTBI subjects (Figure 1) and considering that SLAMF1 appears as a marker of the interaction APCs-T lymphocytes and promotes protection against *Mtb* [18], we hypothesize that MVA including Rv2626c could augment the immunogenicity of the vector.

On the other hand, we previously investigated IL-12 DNA as an adjuvant in an Ag85A DNA prime/MVA85A boost vaccination. We found that vaccination with eukaryotic expression plasmid pCI (pCI)-Ag85A/MVA85A slightly diminished the total CFU per lung, but immunization of mice with pCI-Ag85A + pCI-IL12/MVA85A, significantly decrease the number of CFU per lung as compared to animals that did not receive the adjuvant [15]. In the present work, we evaluated the efficacy of a MVA vaccine vector expressing the mycobacterial dormancy antigen Rv2626c (MVA-Rv2626c) against *Mtb* containment using the same mice model of challenge. Our results showed no differences in CFU in the lungs of MVA-Rv2626c or BCG immunized mice as compared to animals that received PBS (Figure 7). It is important to note that by using Ag85A DNA prime/MVA85A boost vaccination, significant *Mtb* containment in mice lungs was only observed in those animals that also received IL-12 as adjuvant [15] and in our present vaccination schedule with MVA-Rv2626c, no adjuvant was included. Nevertheless, we detected a significant decrease in CFU in the spleens of MVA-Rv2626c vaccinated mice (Figure 7). In contrast, although only 25% of pCI-Ag85A + pCI-IL12/MVA85A vaccinated animals presented bacteria in their spleen, not significant differences were detected as compared to control mice [15]. In summary, the discrepancies observed between our data using MVA85A and our present findings employing MVA-Rv2626c might be associated with several aspects, such as the vaccination schedule and/or the employment of an adjuvant. However, the most relevant difference might be related to the vaccination antigen. In fact, Rv2626c is one of the main genes induced by *Mtb* in response to hypoxia [32] and oxidative stress [33], conditions induced by the granuloma microenvironment [34]. Moreover, Rv2626c is downregulated during TB reactivation [35], suggesting that its expression is limited to the dormancy state. Therefore, since *Mtb* lesions in lungs from mice—unlike those in humans—are not significantly hypoxic [36], the absence of protection in vaccinated mice lungs could be explained by the lack of Rv2626c pulmonary expression. In contrast, the reduction in bacterial burden observed in the spleens of vaccinated mice could result from vaccination limiting bacterial lymphohematogenous dissemination, although the mechanisms underlying this observation remain to be determined. This effect could be explained by Rv2626c induction within the highly inflammatory microenvironment of the lymph node [37], leading to the activation of the immune response against Rv2626c in vaccinated mice.

Contrary to Rv2626c, Ag85A is an early secretory protein heavily secreted during the aerobic replication and logarithmic growth of the bacillus [38]. Unlike latency-associated antigens (such as those regulated by the DosR regulon), the secretion of Ag85A significantly decreases when the bacterium transitions into a dormant, non-replicating state under hypoxic or nutrient-deprived conditions. Due to the high immunogenicity of Ag85A [16], it serves as a cornerstone antigen in the design of next-generation subunit and viral-vectored TB vaccines (such as MVA85A) [39]. Therefore, vaccines including antigens secreted by the pathogen during both active and dormancy stages would be ideal to prevent active disease and reactivation of latent TB. In fact, Rv2626c *Mtb* latency antigen has been previously included in a multistage vaccine [4]. The absence of a significant reduction in pulmonary bacterial burden under the vaccination conditions evaluated here does not exclude the possibility that Rv2626c may contribute to pulmonary protection under optimized vaccination regimens. In particular, the contribution of adjuvant formulations to the protective efficacy of Rv2626c-containing vaccines needs further investigation.

Overall, the present study provides a detailed characterization of the cellular and humoral immunogenic regions of Rv2626c in individuals with LTBI, identifying distinct peptide regions associated with IFN-γ and IgG responses, including a region recognized by both arms of the immune response. The structural mapping of these regions further provides a framework for investigating their contribution to immune recognition. In addition, the reduction in splenic bacterial burden observed following MVA-Rv2626c vaccination provides preliminary experimental support for further investigation of this latency-associated antigen in vaccination strategies. These findings expand our understanding of Rv2626c-specific immune recognition and highlight the value of further exploring its potential contribution, particularly in combination with antigens expressed during different stages of *Mtb* infection, to multistage vaccine approaches.

## 4. Materials and Methods

### 4.1. Study Individuals

BCG-vaccinated healthy adults were recruited for this study. Latent tuberculosis infection (LTBI) was determined using the QuantiFERON-TB Gold In-Tube assay (QFT-GIT; Qiagen, Germantown, MD, USA), performed according to the manufacturer’s instructions (see below). Individuals with a positive QFT-GIT and no clinical or radiological evidence of active TB were classified as having LTBI. All the participants in the LTBI group had a history of previous exposure to TB; however, none had received previous preventive anti-TB treatment before the blood samples were collected. Healthy donors (HD) included adults with no history of TB and negative QFT-GIT results. Subjects with active tuberculosis (TB) were evaluated at Dr. F. J. Muñiz Hospital (Buenos Aires, Argentina). The diagnosis of active TB was established based on clinical and radiological findings together with culture confirmation. None of the patients included in this study had multidrug-resistant (MDR) or extensively drug-resistant (XDR) TB. At the time of enrollment, TB patients had received less than one week of standard anti-TB treatment (rifampicin, pyrazinamide, ethambutol, and isoniazid). Laboratory, radiological and clinical characteristics of enrolled TB patients are displayed in Supplementary Table S4. The demographic characteristics of the three studied groups (HD, LTBI individuals and TB patients) are summarized in Table 1.

All participants provided written informed consent for sample collection and subsequent analyses. The study protocol was approved by the Ethics Committee of Dr. F. J. Muñiz Hospital, and all procedures were conducted in accordance with the relevant guidelines and regulations.

### 4.2. Inclusion and Exclusion Criteria for Study Participants

Inclusion criteria: (i) adults (≥18 years of age) with active pulmonary tuberculosis (TB), and (ii) healthy adult volunteers with no clinical or radiological evidence of active TB. Healthy volunteers were subsequently classified as healthy donors (HD) or individuals with latent tuberculosis infection (LTBI) according to the results of the QuantiFERON-TB Gold In-Tube assay (QFT-GIT), as described below. All participants had received Bacillus Calmette-Guérin (BCG) vaccination. All TB patients had culture-confirmed *Mycobacterium tuberculosis* infection. Exclusion criteria: (i) HIV infection or positive serology for other viral (e.g., hepatitis A, B, or C) or bacterial (e.g., leprosy or syphilis) infections; (ii) diabetes mellitus, cancer, autoimmune diseases, or any other condition that could affect immune function; (iii) pregnancy; and (iv) age < 18 years. Among TB patients, individuals with multidrug-resistant (MDR) or extensively drug-resistant (XDR) TB, and those who had received more than seven consecutive days of anti-TB treatment, were also excluded.

### 4.3. QuantiFERON-TB Gold Plus (QFT) Assay

The QFT (Qiagen, Hilden, Germany; Germantown, MD, USA) test and data interpretation were performed according to the manufacturer´s instructions. QFT results were recorded positive if a single antigen tube (either TB1 or TB2) was ≥0.35 IU/mL as compared to the negative control (“Nil” tube). In TB patients, QFT was performed before the start of antibiotic treatment.

### 4.4. Rv2626c Antigen

Recombinant Rv2626c antigen was produced as previously described [9]. Briefly, Escherichia coli BL21 (DE3) pLysS cells expressing the recombinant protein were cultured, and Rv2626c was purified by nickel affinity chromatography using Ni-NTA agarose (Qiagen, Germantown, MD, USA) according to the manufacturer’s instructions. Protein purity was subsequently verified, and then the preparation was concentrated using Amicon ultrafiltration columns (Merck Millipore, Burlington, MA, USA). Finally, residual endotoxin was removed using Detoxi-Gel Endotoxin Removing Resin (Pierce, Appleton, WI, USA).

### 4.5. Peptides

A set of 36 overlapping peptides spanning the complete Rv2626c sequence was used for peptide-mapping experiments. The peptide design was based on our previous work with Rv2626c and was selected to provide comprehensive coverage of the protein while allowing the identification of immunogenic regions. Peptides were 13–15 amino acids in length and overlapped across adjacent sequences. This strategy was selected because MHC class II-restricted epitopes typically consist of a 9-amino-acid core binding region embedded within longer peptides, making overlapping 13–15-mer peptides suitable for the identification of CD4^+^ T-cell-reactive regions. Peptides were synthesized by GenScript (Nanjing, China) and purity (>90%) was assessed by HPLC. Peptide identity was confirmed by mass spectrometry by the manufacturer. Lyophilized peptides were dissolved in dimethyl sulfoxide (DMSO), aliquoted and stored at −70 °C.

### 4.6. Cell Preparation and Reagents

Peripheral blood mononuclear cells (PBMCs) were isolated by Ficoll-Hypaque density gradient centrifugation (GE Healthcare, Chicago, IL, USA) and cultured at 1 × 10^6^ cells/mL in RPMI 1640 medium (Gibco, Grand Island, NY, USA) supplemented with L-glutamine, penicillin/streptomycin, (Sigma-Aldrich, Burlington, MA, USA) and 10% fetal bovine serum (FBS; Natocor, Córdoba, Argentina). Cells were cultured in the presence or absence of recombinant Rv2626c protein (2.5 μg/mL) or individual Rv2626c-derived peptides (5 μg/mL each). For IFN-γ determination, PBMCs were stimulated for 5 days, after which cell-free supernatants were collected and analyzed by ELISA (Human IFN-γ ELISA MAX™ Standard Kit, BioLegend, San Diego, CA, USA). For flow cytometry experiments, PBMCs were cultured with the corresponding stimuli for 4 days before being harvested for the analysis of cytokine production and surface marker expression, as described below.

### 4.7. Flow Cytometry

After 5 days of PBMCs stimulation, monensin (2 μM; Sigma-Aldrich) was added during the last 5 h of culture. For intracellular cytokine staining, cells were harvested, stained for surface CD4 (300528, BioLegend), fixed with 2% paraformaldehyde (PFA), permeabilized with PBS containing 0.5% (*v*/*v*) saponin (Sigma-Aldrich) and 10% fetal bovine serum (FBS; Natocor), and stained with fluorophore-conjugated antibodies anti-IFN-γ (502512, BioLegend), anti-TNF-α (502906, BioLegend), and anti-IL-2 (500307, BioLegend). In separate experiments, lymphocytes were stained for surface CD3 (300439, Biolegend), SLAMF1 (CD150, 306306, BioLegend) or PD-1 (CD279, 621608, Bio-Legend). As a positive control for flow cytometry assays, PBMCs were stimulated with whole-cell lysate from the virulent *Mycobacterium tuberculosis* H37Rv strain (*Mtb*-Ag; BEI Resources, NIAID, NIH). Samples were acquired on a FACSAria II flow cytometer (BD Biosciences, San Jose, CA, USA).

For flow cytometry analysis, isotype-matched controls were included for both the unstimulated (medium) and positive control conditions. The isotype control from the positive control condition was used to establish the positivity threshold for each intracellular cytokine and the same gates were applied to antigen-stimulated condition. Cytokine-positive CD4^+^ T-cell frequencies were then determined for each condition. To account for background cytokine production, the frequency of cytokine-positive CD4^+^ T cells detected in the unstimulated medium control was subtracted from the corresponding stimulated condition.

Boolean gate analysis was subsequently performed using FlowJo™ v10 software to determine the frequencies of CD4^+^ T cells producing IFN-γ, IL-2, and TNF-α either individually or in combination. This analysis allowed the identification of mono-, double-, and triple-cytokine-producing CD4^+^ T cells. In particular, CD4^+^ T cells simultaneously expressing IFN-γ, IL-2, and TNF-α were defined as triple-positive T cells.

### 4.8. Determination of Plasma IgG Antibodies Against Rv2626c-Derived Peptides by ELISA

Plasma IgG responses against individual Rv2626c-derived synthetic peptides were determined by ELISA. High-binding 96-well plates were coated overnight with each peptide at a concentration of 2.5 μg/mL, leaving uncoated wells as control for unspecific binding. Plates were then blocked with 1% bovine serum albumin (BSA) in phosphate-buffered saline. Plasma samples, obtained from heparinized unstimulated whole blood, were diluted 1:100 and assayed in duplicate. After incubation with the samples, bound IgG was detected using a biotin-conjugated mouse anti-human IgG antibody (BD Pharmingen, San Diego, CA, USA), followed by high-sensitivity streptavidin–horseradish peroxidase (Thermo Fisher Scientific, Waltham, MA, USA). The reaction was developed with 3,3′,5,5′-tetramethylbenzidine (TMB) substrate (Sigma-Aldrich, USA) for 30 min, stopped with 2 N sulfuric acid, and absorbance was measured at 450 nm. Finally, absorbance from uncoated wells was subtracted from peptide-coated wells. For the exploratory analysis of peptide-specific IgG responses, a general positivity threshold was established as the mean IgG reactivity of 20 healthy donors plus 3 standard deviations (SD). A single general threshold was applied rather than deriving an independent cutoff for each peptide, as the analysis was intended to be exploratory rather than to establish peptide-specific diagnostic thresholds.

### 4.9. Structural Modeling and Peptide Mapping

The structure of Rv2626c was analyzed using YASARA [14]. The complete Rv2626c, including residues 124–143, which are absent in the X-ray structure (PDB ID: 1Y5H) [40], was modeled using AlphaFold v3 [41], and the resulting model was compared with the experimental structure, showing high structural superimposition. The above structural alignment has a Cα RMSD of 0.88 Å across 122 aligned residues with 100% sequence identity. The AlphaFold v3 model was used for the analysis and to map the detected peptides by the immune responses.

### 4.10. Generation of Recombinant MVA-Rv2626c

The recombinant Modified Vaccinia virus Ankara expressing the Rv2626c antigen (MVA-Rv2626c) was generated by homologous recombination using the infection/transfection method. Briefly, the complete coding sequence of Rv2626c was cloned into an MVA transfer vector under the control of a synthetic early/late vaccinia virus promoter and flanked by sequences homologous to the viral thymidine kinase (TK) locus. Primary chicken embryo fibroblasts infected with wild-type MVA were transfected with the recombinant transfer plasmid, and recombinant viruses were isolated by plaque purification under agar overlay using β-glucuronidase activity as the selection marker. Following multiple rounds of plaque purification, homogeneous recombinant viral stocks were obtained. The identity of the recombinant virus was confirmed by PCR amplification of the Rv2626c insert and disruption of the viral TK locus, whereas transgene expression was verified by Western blot and immunofluorescence.

### 4.11. Animals, Immunization and Challenge

Specific-pathogen-free female BALB/c mice, aged 6 to 8 weeks, were obtained from the Central Animal Facility of the Faculty of Exact and Natural Sciences at the University of Buenos Aires. The animals were housed in a biosafety level 3 (BSL-3) facility in accordance with approved procedures (Approval code: DEP-20-00). Mice were kept in ventilated racks with ad libitum access to balanced feed and water. All experimental procedures were conducted in accordance with international standards for the use and care of laboratory animals [42,43], aiming to minimize animal suffering. Animals were randomly allocated to the different treatment groups. Mice received two sublingual (s.l.) immunizations, spaced two weeks apart, with MVA-Rv2626c (1 × 10^5^ CFU per dose; *n* = 7). As negative and positive controls, animals were immunized sublingually with PBS (*n* = 6) or subcutaneously with *Mycobacterium bovis* BCG Danish; 5 × 10^4^ CFU; *n* = 5), respectively. Thirty days after the final immunization, the animals were challenged intratracheally with the pathogenic *Mycobacterium tuberculosis* strain H37Rv (1 × 10^4^ CFU). *Mtb* H37Rv strain was grown in Middlebrook 7H9 broth (BD Biosciences) or in 7H10 agar (BD Biosciences), 0.2% (*v*/*v*) glycerol, and albumin–dextrose–catalase–oleic acid supplement (BD Biosciences). Cultures were harvested at exponential growing phase at 37 °C and clumps were disaggregated by glass bead vortexing and passage through a 25 Gauge needle. Bacteria were centrifuged twice for 10 min at 3300× *g* and supernatants were diluted with physiological solution. Finally, O.D. at 600 nm was determined. Thirty days post-challenge, the animals were euthanized, and the spleen and lungs were aseptically removed to determine *Mtb* colony-forming units per milliliter (CFU). Whole organs were homogenized in 1 mL of sterile PBS. Then, serial dilutions (triplicates) were prepared, and 10 μL of each dilution was plated in Middlebrook 7H10 agar for CFU counting. Bacterial burden was assessed by investigators blinded to the study groups.

### 4.12. Sample Size and Statistical Analysis

The number of samples varied among individual flow-cytometry experiments. Specifically, *n* = 5–11 individuals per group were analyzed for IFN-γ^+^ CD4^+^ T cells, *n* = 6–7 per group for SLAMF1 expression, *n* = 5–11 per group for PD-1 expression, and *n* = 7 per group for polyfunctional IFN-γ^+^TNF-α^+^IL-2^+^ CD4^+^ T cells. For the peptide-specific IFN-γ analysis, the sample size was determined based on the exploratory nature and experimental complexity of the peptide-mapping study. The objective was to characterize the immune response to 36 overlapping Rv2626c-derived peptides spanning the entire protein and to identify candidate immunodominant regions based on both IFN-γ response magnitude and responder frequency. The number of individuals analyzed for each peptide was determined based on the feasibility of performing the complete peptide-mapping analysis while ensuring sufficient data to identify patterns of peptide recognition. The number of LTBI individuals analyzed for each peptide was as follows: *n* = 4 for peptides 6, 18, 19, 26, 27, 28, and 29; *n* = 5 for peptides 13, 14, 15, 16, 17, 20, 21, 22, and 30; *n* = 6 for peptides 1, 2, 5, 7, 8, 9, 11, 24, 25, 32, and 34; *n* = 7 for peptides 3, 4, 10, 12, 23, and 36; and *n* = 9 for peptides 31, 33, and 35. Peptide-specific IgG responses were evaluated using plasma samples from eight individuals with LTBI. Antibody responses and responder frequencies were also evaluated descriptively. For the in vivo vaccination and challenge experiment, seven mice per group were included. The sample size was established prospectively as part of the animal study protocol approved by the Institutional Committee for the Care and Use of Laboratory Animals (CICUAL). Prior to statistical comparisons, the distribution of the data and the assumptions underlying the corresponding statistical tests were assessed through formal tests and visual inspection of Q–Q plots. Parametric or non-parametric tests were then selected depending on whether the data met the assumptions required for normality. For comparisons involving flow-cytometry data, which did not meet the assumptions for parametric analysis, the Kruskal–Wallis test followed by Dunn’s multiple-comparisons test with Bonferroni correction was used. For the in vivo vaccination/challenge experiment, the data fulfilled the assumptions for parametric analysis (following log transformation) and comparisons were therefore performed using one-way ANOVA followed by Tukey’s multiple-comparisons test. Analyses were performed using GraphPad Prism 10.2 software and *p* < 0.05 was considered statistically significant.

## Figures and Tables

**Table 1:** Demographic characteristics of the study participants. The number of individuals included in the study is specified for healthy donors (HD), individuals with latent tuberculosis infection (LTBI) and active TB patients (TB). For age, values are represented as mean ± SEM.

		HD	LTBI	TB
Number (*n*)		24	37	14
Age (years)		39.3 ± 3.7	33.1 ± 2.3	37.1 ± 4.4
Sex	Female	70%	75%	14%
	Male	30%	29%	86%
Country of origin	Argentina	86%	82%	69%
	South America (others)	14%	18%	31%
Exposure to *Mtb*	<1 year	-	24%	-
	>1 year	-	76%	-

## Data Availability

All data generated or analyzed during this study are included in this published article (and its Supplementary Information Files).

## Supplementary Materials

The following supporting information can be downloaded at: https://www.mdpi.com/article/10.3390/ijms27177827/s1.

## Abbreviations

The following abbreviations are used in this manuscript:

APC	Antigen Presenting Cells
CFU	Colony-Forming Units per milliliter
CMI	Cell Mediated Immunity
HD	Healthy Donors
LTBI	Individuals with Latent Tuberculosis Infection
*Mtb*	*Mycobacterium tuberculosis*, *M. tuberculosis*
MVA	Modified Vaccinia Ankara
MVA85A	Modified Vaccinia Ankara vector expressing Ag85A
MVARv2626c	Modified Vaccinia Ankara vector expressing Rv2626c
PBMCs	Peripheral Blood Mononuclear Cells
PBS	Phosphate-Buffered Saline
pCI-Ag85A	Recombinant eukaryotic expression plasmid encoding Ag85A
pCI-IL-12	Recombinant eukaryotic expression plasmid encoding IL-12
PD-1	Programmed Cell Death Protein 1
PD-L1	Programmed Death-Ligand 1
TFH	T follicular helper cell
s.l.	Sublingual immunization
SLAMF1	Signaling Lymphocytic Activation Molecule Family Member 1
TB	Tuberculosis
