# Supplementary material for: Targeting Latent Tuberculosis: Immunogenic Evaluation of a Mycobacterium tuberculosis Dormancy Antigen as a Vaccine Candidate
Source: Int J Mol Sci. 2026 Sep 1;27(17):7827. doi: 10.3390/ijms27177827 (PMC13565904; doi:10.3390/ijms27177827)
Supplement: Supplementary file 1 [file ijms-27-07827-s001.zip › ijms-4492952-supplementary.pdf]

Supplementary Section

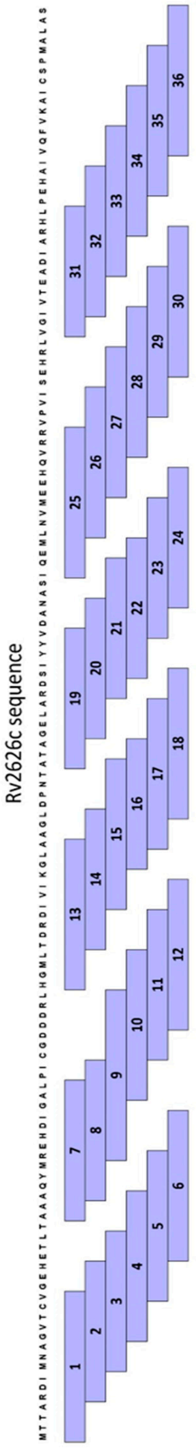

Supplementary Figure S1. Schematic representation of overlapping synthetic peptides spanning the entire Rv2626c sequence. Amino acid sequence of Rv2626c and its 36 overlapping peptides (numbered 1 to 36) is shown. The peptides, ranging from 13 to 15 amino acids in length and overlapping by 11 amino acids, cover the whole Rv2626c sequence.

Supplementary Table S1. Amino acid sequences of the synthetic overlapping peptides spanning the Rv2626c protein.

Peptides	Amino acid sequence
1	MTTARDIMNAGVTCV
2	RDIMNAGVTCVGEH
3	MNAGVTCVGEHETL
4	GVTCVGEHETLTAAA
5	VGEHETLTAAAQYMR
6	ETLTAAAQYMREHDI
7	AAAQYMREHDIGAL
8	AQYMREHDIGALPI
9	MREHDIGALPICGDDDR
10	GALPICGDDDRLHGM
11	ICGDDDRLHGMLTDR
12	DDRLHGMLTDRDIVI
13	HGMLTDRDIVIKGLA
14	TDRDIVIKGLAAGL
15	DIVIKGLAAGLDPNTA
16	GLAAGLDPNTATA
17	AAGLDPNTATAGELA
18	DPNTATAGELARDSI
19	ATAGELARDSIYYV
20	GELARDSIYYVDANA
21	RDSIYYVDANASI
22	SIYYVDANASIQEML
23	VDANASIQEMLNVM
24	NASIQEMLNVMEEH
25	IQEMLNVMEEHQVRR
26	LNVMEEHQVRRPVI
27	EEHQVRRVPVISEHR
28	VRRVPVISEHRLVGI
29	PVISEHRLVGIVTEA
30	EHRLVGIVTEADIAR
31	VGIVTEADIARHL
32	IVTEADIARHLPEHA
33	ADIARHLPEHAIVQF
34	RHLPEHAIVQFVKAI
35	EHAIVQFVKAICSPM
36	VQFVKAICSPMALAS

Supplementary Table S2. Experimental design and immune response following mice immunization. BALB/c mice were initially immunized with two doses of MVA2626c (1×10^5 CFU) administered via the sublingual (s.l.) route, 14 days apart. Ten days after the last immunization (Figure 4, original manuscript), the animals were sacrificed, and splenocytes from vaccinated mice were stimulated *in vitro* with peptide pool F derived from Rv2626c (2 µg/mL) for 4 h (Flow cytometry) or 72h (ELISA). Afterwards, IFN-γ production was determined. Data are presented as the mean (subtracting the unstimulated condition) of the %$CD4^+$IFN-γ^+ cells ± SEM or the mean of IFN-γ production (pg/mL) (subtracting the unstimulated condition) ± SEM. Data normality was assessed using a normality test. Since the data followed a normal distribution, comparisons between two independent groups were performed using an unpaired two-tailed Student's t-test.

Measurement	PBS	MVA-Rv2626c	*p*-value
**%$CD4^+$ IFNγ^+ cells**	0,05 ± 0,03	7,45 ± 0,1732	<0,0001
**IFNγ (pg/mL)**	1,581 ± 0,05	3,465 ± 0,05398	<0,0001

Supplementary Table S3. Protective efficacy of MVA-Rv2626c vaccination against *M. tuberculosis* challenge. BALB/c mice were immunized via the sublingual route with PBS (n=6), BCG (n=5), MVA-Rv2626c (n=7) and subsequently challenged with virulent *M. tuberculosis*. Bacterial burden for each mice/organ was determined by quantifying *M. tuberculosis* colony-forming units per milliliter (CFU) by triplicate and is expressed as $\log_{10}$ Mean ± SD. Log protection was calculated relative to the PBS control group as $\log_{10}$ CFUcontrol − $\log_{10}$ CFUvaccinated, where the PBS group was considered the control group. Statistical significance was assessed by one-way ANOVA followed by Dunnett's multiple-comparisons test, comparing each treatment group with the PBS control group. [a] *p*-value <0,01 compared with the PBS control group.

Treatment group	Vaccine	Log_{10} *Mtb* CFU (mean ± SD) at:		Log protection at:	
		Lung	Spleen	Lung	Spleen
1	PBS	5,20 ± 0,098	4.62 ± 0,290	-	-
2	BCG	4,95 ± 0,200	3.93 ± 0,143	0,25	0,69[a]
3	MVA-Rv2626c	5,22 ± 0,162	3,76 ± 0,119	- 0,02	0,86[a]

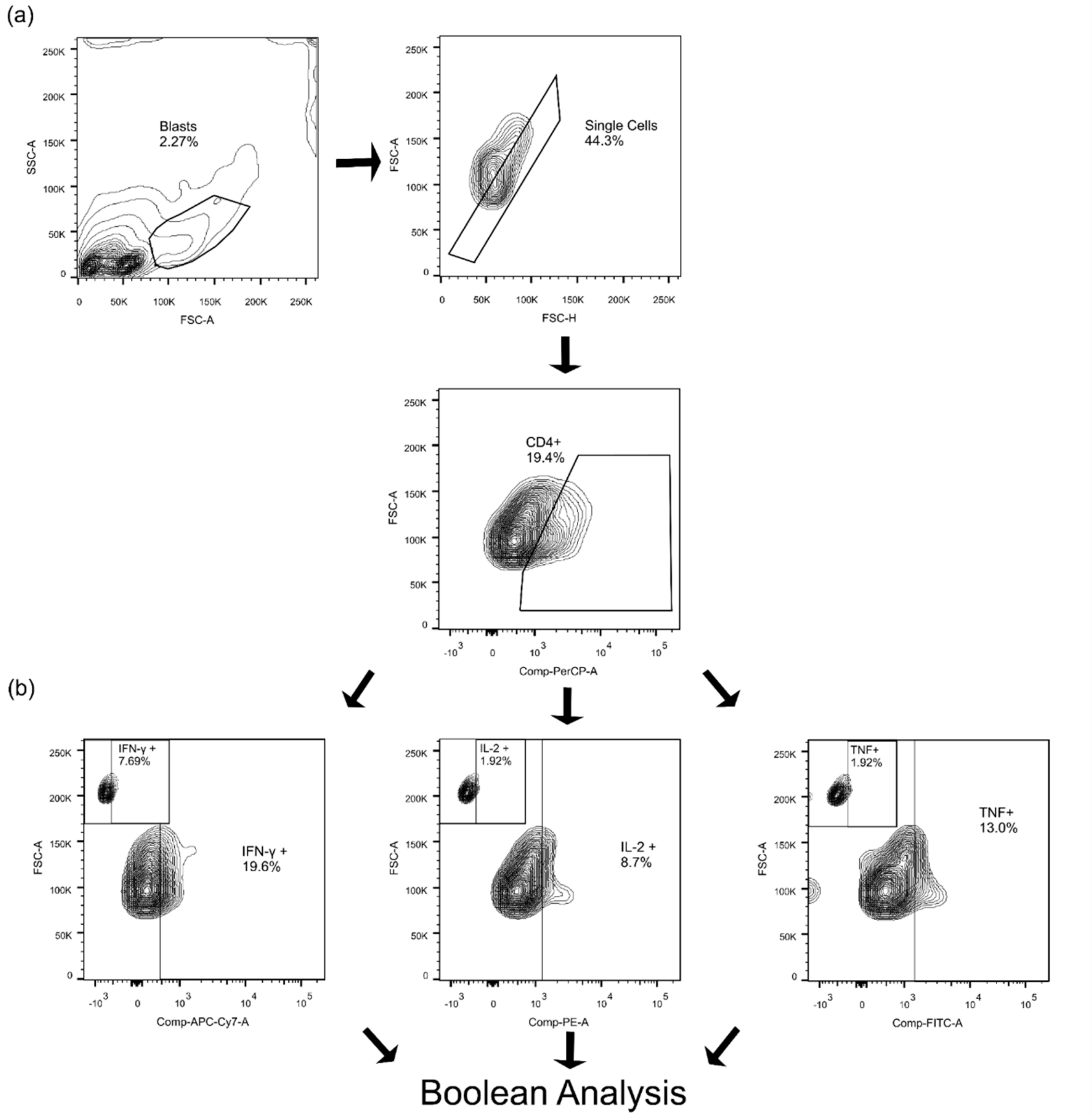

Supplementary Figure S2. Gating strategy for the identification of IFN-γ-producing and triple-positive $CD4^+$ T cells. Representative flow cytometry plots illustrating the gating strategy used to identify cytokine-producing $CD4^+$ T cells following Rv2626c stimulation in an LTBI individual. (A) Cells were first gated based on FSC-A and SSC-A to identify the blast population, followed by singlet discrimination using FSC-A versus FSC-H. $CD4^+$ cells were subsequently identified within the selected population. (B) IFN-γ-, IL-2-, and TNF-α-producing $CD4^+$ cells were identified using the corresponding cytokine gates. Cytokine positivity gates were established using the corresponding isotype controls from the positive control condition and subsequently applied to the stimulated samples. The frequencies of cytokine-producing cells were then analyzed by Boolean gating to determine the different combinations of IFN-γ, IL-2, and TNF-α production, including mono-, double-, and triple-cytokine-producing $CD4^+$ cells.

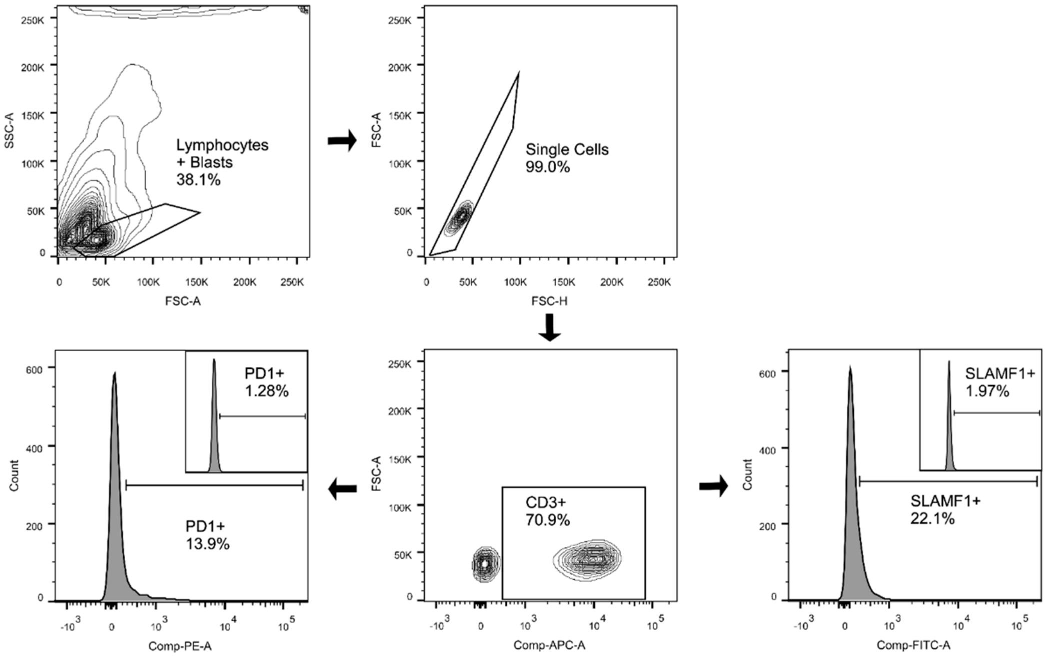

Supplementary Figure S3. Gating strategy for the identification of PD-1- and SLAMF1-expressing T cells. Representative flow cytometry plots illustrate the strategy used to identify PD-1- and SLAMF1-expressing T cells following stimulation with Rv2626c in an LTBI individual. Lymphocytes and blasts were first identified based on FSC-A and SSC-A characteristics, followed by singlet discrimination using FSC-A versus FSC-H. $CD3^+$ T cells were subsequently identified within the singlet population. The expression of PD-1 and SLAMF1 was then assessed by applying the corresponding positivity gates to the $CD3^+$ T-cell population. Representative histograms show the gates used to determine $PD\text{-}1^+$ and $SLAMF1^+$ cells. The frequency of PD-1- and SLAMF1-positive cells was corrected for background by subtracting the corresponding frequency observed in the unstimulated negative control.

Supplementary Table S4. Characteristics of active TB patients. Laboratory, radiological and clinical characteristics of active TB patients included in this study are detailed. AFB smear results were semi-quantitatively graded as +++ (>200 bacilli within 20 fields), ++ (50-500 bacilli within 50 fields), + (10-99 bacilli within 100 fields) or - (<9 bacilli within 100 fields); according to standard smear microscopy grading criteria. Pulmonary compromise and presence of cavitary lesions was determined by expert radiologists through evaluation of chest x-ray images. For continuous variables, values are represented as mean ± SEM.

		**TB**
Symptom onset (months)		2,97 ± 0,74
Bacillary load	Low (-/+)	43%
	High (++/+++)	57%
Pulmonary compromise	Unilateral	33%
	Bilateral	64%
Cavitary TB		79%
Albumin (g/dl)		3,15 ± 0,26
Leucocytes (cells/mm^3)		11481.82 ± 969.80

Lymphocytes (cells/mm^3)	1752.63 ± 285.54
Neutrophils (cells/mm^3)	7512.33 ± 794.18
Monocytes (cells/mm^3)	944.36 ± 198.53
Neutrophil-to-lymphocyte ratio	0.93 ± 0.26
